# Prevention of seasonal affective disorder in daily clinical practice: results of a survey in German-speaking countries

**DOI:** 10.1186/s12888-017-1403-2

**Published:** 2017-07-11

**Authors:** B. Nussbaumer-Streit, D. Winkler, M. Spies, S. Kasper, E. Pjrek

**Affiliations:** 1 Department of Psychiatry and Psychotherapy, Medical University of Vienna, Währinger Gürtel 18–20, 1090 Vienna, Austria; 2 Department for Evidence-based Medicine and Clinical Epidemiology, Danube-University Krems, Dr.-Karl-Dorrek Strasse 30, 3500, Krems a.d. Donau, Austria

**Keywords:** Seasonal affective disorder, Winter depression, Prevention, Austria, Germany, Switzerland

## Abstract

**Background:**

Seasonal affective disorder (SAD) is a seasonally recurrent type of major depression. This predictable aspect makes it promising for preventive treatment. However, evidence for the efficacy and harm of preventive treatment of SAD is scarce, as are recommendations from clinical practice guidelines. The aim of this study was to assess the current use of preventive treatment of SAD in clinical practice in German-speaking countries for the first time.

**Methods:**

We conducted a postal and web-based survey sent to the heads of all psychiatric institutions listed in the inventory “Deutsches Krankenhaus Adressbuch, 2015” that contains all psychiatric hospitals in Germany, Austria, and Switzerland.

**Results:**

One hundred institutions (out of 533 institutions, 19%), which treated in total more than 3100 SAD patients in the years 2014/2015, responded. Of those, 81 reported recommending preventive treatment to patients with a history of SAD. There was no consensus on the optimal starting point for preventive treatment. Most of the institutions that implemented prevention of SAD, recommended lifestyle changes (85%), antidepressants (84%), psychotherapy (73%), and light therapy (72%) to their patients. The situation was similar in northern and southern regions.

**Conclusions:**

Most hospitals recommended the use of preventive treatment to SAD patients, although evidence on efficacy and harm is limited. A wide variety of interventions were recommended, although guidelines only include recommendations for acute treatment. To assist psychiatrists and patients in future decision making, controlled studies on preventive treatment for SAD that compare different interventions with one another are needed.

## Background

Seasonal affective disorder (SAD), winter-type, is a subtype of major depression, characterized by a recurrent pattern in which depressive episodes start in fall/winter and remit in spring [1, 2]. It affects 2–8% of the total population in Europe, depending on latitude [3–7]. In German-speaking countries approximately 2.5% of the population suffers from SAD [8, 9]. About 80% of those diagnosed with this illness will face a recurrent depressive episode the following winter [10], which has detrimental effects on their quality of life. Five to 11 years following initial diagnosis, SAD resolves in only 14–18% of patients, persists in 22–42%, and develops into a non-seasonal major depression in 33–44% of patients [11, 12].

Since decreased seasonal exposure to environmental light is thought to be a trigger for developing SAD, light therapy is the first line of treatment [13]. At the neurochemical level, changes in both the serotonergic and catecholaminergic transmitter systems seem to play key roles in SAD [14]. Therefore, antidepressants have become the second choice of treatment. Of these, only one (bupropion extended release [XL]) is currently approved for the prevention of SAD [15]. Treatment options other than light therapy and antidepressants include agomelatine [16], melatonin [17], psychological interventions [18–21], as well as lifestyle and diet changes [22].

The seasonal recurrence of depressive episodes provides the rationale for these interventions as a preventive treatment of SAD [23]. Implementing them in symptom-free periods could avoid the development of new depressive episodes in the next fall/winter. However, very little is known about the efficacy and potential harms of preventive treatments in SAD patients. A recent systematic review demonstrated that the preventive use of the antidepressant bupropion XL reduced the number of male and female patients developing a new depressive episode in the next winter by 44% compared to placebo [24]. An additional systematic review on preventive light therapy in SAD patients identified only one study with high risk of bias, allowing no valid conclusions [25]. A systematic review on agomelatine, and melatonin was not able to identify any randomized controlled trials [26]. A recent randomized controlled trial on preventive psychotherapy showed that cognitive behavioural therapy led to lower recurrence of SAD-episodes in the following winters compared to light therapy (27.3% compared to 45.6%) [27].

The German clinical practice guideline on depression recommends the use of light therapy for SAD patients during the period of risk (fall/winter), but not during the summer months [28]. No other recommendations regarding prevention of SAD episodes are provided. Thus, clinicians are forced to rely on recommendations for patients with a non-seasonal major depression. For prevention of recurrent depressive episodes in these patients, continuation treatment with antidepressants and psychotherapy is recommended [28]. Despite much uncertainty, patients and clinicians may decide for preventive interventions in daily clinical practice. To date, however, there is no published evidence to what extent preventive interventions are used, or which interventions are used for this purpose, if any. The objective of our study was to determine clinical practice patterns regarding the prevention of SAD episodes in German speaking countries.

## Methods

To achieve our objective, we employed an international survey in German-speaking countries. The study was approved by and registered with the ethical review board of the Medical University of Vienna (EC No 1586/2015).

### Sampling frame

We used the inventory “Deutsches Krankenhaus Adressbuch, 2015” [29] that contains all psychiatric institutions in German speaking countries as our sampling frame. We removed 58 institutions, after validation via an internet-search, because they had been closed, merged with other psychiatric institutions, or exclusively provided child and adolescent psychiatric care. Thus, the basic survey population comprised 533 psychiatric hospitals and departments in Austria, Germany, and Switzerland.

### Survey method

We developed a questionnaire in the German language according to established standards [30] and partly based on a questionnaire used in a former study on the use of light therapy [31]. We pre-tested the questionnaire with four psychiatrists working at the Medical University of Vienna and checked face-validity within our research team. The questionnaire was structured into a general part on acute treatment of SAD and a specific part on preventive treatment of SAD. The first part included one question on the number of patients treated for SAD in the institution and one assessing what interventions were used to treat acute depressive episodes in SAD patients. We asked about the use of light therapy, antidepressants, agomelatine, melatonin, methylphenidate, psychotherapy, lifestyle changes, diet changes, and alternative approaches. We named examples of lifestyle changes (e.g. sports, brighter homes), diet changes (e.g. vitamin D, no caffeine intake), and alternative approaches (e.g. meditation, yoga, acupuncture) to give an idea of what type of interventions were included in this category.

In the second part we asked if hospitals recommend interventions to prevent the onset of a new depressive episode in the next fall/winter in patients with a history of SAD and what timing they prefer for preventive treatment. We explicitly asked if light therapy, antidepressants, agomelatine, melatonin, methylphenidate, psychotherapy, lifestyle changes, diet changes, and alternative approaches were used as preventive treatment, and if so in how many patients, and for how long. We also asked participants to name the specific type of intervention used; e.g. the name of the antidepressants used for preventive treatment of SAD. Head of participating departments and hospitals were asked to use the years 2014/2015 as reference periods and to reflect the policies in their hospitals rather than their individual opinion in their answers.

We conducted the survey between December 2015 and April 2016. First, we sent a postal questionnaire accompanied by a cover letter and a stamped response-envelope to the heads of all psychiatric departments and hospitals from our survey population. In order to reach out to non-responders we set up a web-based survey based on the questionnaire with the software SurveyMonkey [32] in February 2016. We sent a survey-invitation to all non-responders as well as two reminder e-mails, one in March 2016, and one in April 2016, and called them once per phone.

### Analysis

We analysed responses mainly using descriptive statistics. Main outcomes of interest were 1) the proportion of departments and hospitals recommending preventive treatment to SAD patients, 2) the timing of preventive treatment, 3) the frequency of different interventions recommended for preventive treatment. In addition, we wanted to know the proportion of patients receiving, as well as the timing and specification of, each preventive treatment (e.g. type of antidepressant).

Switzerland, Austria, and Germany have only a small latitude cline (47°-54°), but differences in sunshine hours during winter, e.g. in February more than 100 sunshine hours in Austria and in the south of Germany compared to less than 50 h in the north of Germany [33, 34]. We assumed that this might correlate with greater demand for preventive treatment in northern regions. In our analysis Austria, Switzerland, Bavaria, and Baden-Württemberg comprised the southern region, and all other states of Germany were defined as the northern part. We also hypothesised that different health care systems and reimbursement procedures might lead to different patterns of recommendations between countries as well as the type of hospital. To explore differences in preventive treatments recommended between northern and southern regions, countries, and type of hospital, we a priori defined these subgroups. We tested for differences between these subgroups by using Fisher’s Exact Test, with a level of significance of *p* ≤ 0.05 for all tests, without correction for multiple-comparisons. All calculations were completed in IBM SPSS Statistics Version 24, Armonk, NY [35].

## Results

### Respondents

In total, 100 hospitals participated in the survey. Of these, 18 were from Austria, 71 from Germany, and 11 from Switzerland. This is reflective of a 19% (100/533) response-rate. These questionnaires summed up experience in 3.119 SAD patients over a two-year period (2014/2015). For further details on participating hospitals see Table 1.

**Table 1:** Characteristics of participating hospitals

Characteristics	Number of hospitals (response in %)
Country (*n* = 100)	
Austria	18 (18%)
Germany	71 (71%)
Switzerland	11 (11%)
Type of hospital (*n* = 99)	
University hospital	21 (21%)
Teaching hospital	23 (23%)
Specialized psychiatric hospital	33 (33%)
Psychiatric department in general hospital	22 (22%)
Patients treated because of acute SAD in 2014/2015 (*n* = 92)	
0% SAD patients treated 2014/2015	14 (15%)
≤ 5% of patients treated because of SAD	58 (63%)
5,1%–10% of patients treated because of SAD	13 (14%)
≥ 10% of patients treated because of SAD	5 (5%)
Interventions used to treat acute SAD (*n* = 86)	
Antidepressants	85 (99%)
Lifestyle changes	78 (91%)
Light therapy	75 (87%)
Psychotherapy	73 (85%)
Agomelatine	61(71%)
Dietary changes	48 (56%)
Alternative methods (e.g. meditation)	47 (55%)
Melatonin	17 (20%)
Methylphenidate	5 (6%)

n, total number of participating hospitals that answered this question; SAD, seasonal affective disorder

### Proportion of hospitals recommending preventive treatment and timing

The majority (84%) of the 96 psychiatric hospitals and departments that answered this question recommend the use of preventive interventions in order to reduce the risk of the onset of a new depressive episode in the upcoming fall/winter season in patients with a history of SAD. There is no consensus, however, on the optimal timing for preventive treatment in general: 38% (*n* = 81) recommend continuing acute treatment of SAD throughout the summer, 31% (*n* = 81) recommend to their patients to start with the preventive intervention as long as they are in remission and before depressive symptoms start (i.e. at end of summer), and 14% (*n* = 81) tell their patients to start with preventive treatment as soon as they feel mild depressive symptoms. The remaining institutions (17%, *n* = 81) use all three approaches, depending on the type of preventive treatment.

### Types of interventions recommended for preventive treatment

**Fig. 1:**
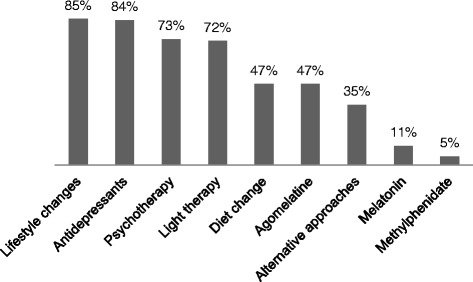
Percentage of hospitals recommending types of SAD prevention (*n* = 81)

The most common types of recommended preventive interventions are lifestyle changes and antidepressants (Fig. 1).

Eighty-five percent of hospitals and departments (69/81) consider lifestyle changes a viable preventive treatment option. Those who recommend lifestyle adjustment, suggest it on average to 83% of their patients with a history of SAD. More than half of institutions recommend a permanent lifestyle change. Suggested lifestyle changes comprise exercise on a regular basis, exercise outdoors, spending time outdoors in the sunlight, striving towards a regular sleep-wake cycle, partaking in enjoyable activities (e.g. hobbies, meeting friends), spending their winter vacations in sunny regions, and trying to redecorate the home to make it brighter (Table 2).

**Table 2:** Details on type, frequency, and timing of preventive interventions recommended in clinical practice

Preventive intervention	Hospitals recommending intervention	Average percentage of SAD patients being given this recommendation (min-max)	Timing of preventive intervention	Type of preventive intervention recommended (multiple mentions possible)
Lifestyle changes	85% (69/81)	83% (10%–100%)	• 12% (8/69) recommend starting with lifestyle changes by the end of summer for 4–32 weeks • 64% (44/69) recommend continuous lifestyle changes throughout the whole year • 24% (17/69) no response	Based on 69 hospitals recommending lifestyle changes: • Regular exercise (41%) • Regular exercise outdoor (14%) • Do things you like (hobbies, meeting friends) (13%) • Find ways to relax (14%) • Sleep hygiene (13%) • Ensure stable day/night rhythm (13%) • Spending time outdoor (9%) • Structured lifestyle (7%) • Redecorate rooms to make them brighter (4%) • Winter vacation in a sunny region (3%)
Antidepressants	84% (68/81)	70% (10%–100%)	• 21% (14/68) recommend starting preventive treatment by the end of summer for 4–28 weeks • 62% (42/68) recommend continuous intake of antidepressants throughout the whole year • 17% (12/68) no response	Based on 68 hospitals recommending antidepressants: *Selective serotonin reuptake inhibitors (SSRI)* • Citalopram (10%), Escitalopram (12%), Sertraline (10%) • Not further specified (40%) *Selective serotonin and norepinephrine reuptake inhibitors (SSNRI)* • Venlafaxine (22%), Duloxetine (4%), Milnacipran (1%) • Not further specified (19%) *Monoamine oxidase inhibitors (MAO-H)* • Moclobemide (1%), Not further specified (6%) *Noradrenergic and specific serotonergic antidepressant (NaSSA)* • Mirtazapine (19%), Not further specified (6%) *Serotonin antagonist and reuptake inhibitor (SARI)* • Trazodone (3%) *Norepinephrine and dopamine reuptake inhibitor (NDRI)* • Bupropion (7%) *Tryclic antidepressant (TZA)* • Amitriptyline (3%) *Serotonin modulators and stimulators:* • Vortioxetine (3%)
Psychotherapy	73% (59/81)	62% (10%–100%)	• 22% (13/59) recommend starting preventive psychotherapy by the end of summer for 1–30 weeks • 41% (30/59) recommend continuous psychotherapy throughout the whole year • 27% (16/59) no response	Based on 59 hospitals recommending psychotherapy: • Behavioural therapy (32%) • Analytic psychotherapy (10%) • Psychotherapy not otherwise specified (7%) • Talking therapy (3%) • Psychoeducation (2%) • Family therapy (2%) • Hypnotherapy (2%) • Systemic therapy (2%)
Light therapy	72% (58/81)	64% (10%–100%)	• 47% (27/58) recommend to start preventive light therapy by the end of summer for 3–16 weeks • 31% (18/58) recommend continuous use of light therapy throughout the whole year • 22% (13/58) no response	Based on 58 hospitals recommending light therapy: • Light therapy device with 10,000 lx (40%) • Spending time in natural sunlight (12%) • Light therapy device with 6000 lx (3%) • Light therapy device with 2000 lx (2%) • Light therapy device with 200 lx (2%) • Infrared light (2%) • Light visor (2%) • “Light shower” (2%)
Diet change	47% (38/81)	71% (10%–100%)	• 11% (4/38) recommend to start diet changes by the end of summer for 8–26 weeks • 61% (23/38) recommend continuous diet change throughout the whole year • 29% (11/38) no response	Based on 38 hospitals recommending diet changes: • Balanced diet, e.g. Mediterranean diet, less carbohydrates, more fibres, less meat (47%) • Less coffee (24%) • Less nicotine (11%) • Less alcohol (11%) • Vitamin D (8%) • No heavy meals in the evenings (8%) • Nutritional Supplements, e.g. Vitamin B12, iron (5%) • Increased fluid intake (3%)
Agomelatine	47% (38/81)	24% (10%–70%)	• 21% (8/38) recommend to start by the end of summer for 4–36 weeks • 58% (22/38) recommend continuous intake • 21% (8/38) no response	Based on 38 hospitals recommending agomelatine: • Agomelatine (66%)
Alternative approaches	35% (28/81)	57% (10%–100%)	• 18% (5/28) recommend to start preventive alternative treatments by the end of summer for 4–32 weeks • 68% (19/28) recommend continuous treatment throughout the whole year • 14% (4/28) no response	Based on 28 hospitals recommending alternative treatments: • Yoga (29%) • Relaxation techniques (29%) • Acupuncture (21%) • Meditation (14%) • Progressive muscle relaxation (14%) • Homeopathy (4%) • Aroma therapy (4%) • Sleep deprivation (4%) • Kinesiology (4%) • Tai Chi (4%) • Chi Gong (4%) • Shiatsu (4%) • Reiki (4%)
Melatonin	11% (9/81)	23% (10%–70%)	• 78% (7/9) recommend to start by the end of summer for 3–30 weeks • 11% (1/9) recommend continuous treatment • 11% (1/9) no response	Based on 9 hospitals recommending melatonin: • Melatonin (33%)
Methylphenidate	5% (4/81)	10% (10%–10%)	• 1 institution recommends preventive treatment for 12 weeks • 2 institutions recommend continuous treatment • 1 institution did not respond	Based on 4 hospitals recommending methylphenidate • Methylphenidate (50%)

Max, highest percentage named; min, lowest percentage named; n, total number of hospitals answering this question; SAD, seasonal affective disorder

Most departments and hospitals also consider antidepressants an acceptable preventive treatment (68/81). On average they recommend antidepressants to 70% of their SAD patients. Institutions named a variety of antidepressants that they prescribe as preventive treatment (Table 2). Bupropion XL, the only medication licensed for prevention in SAD patients, is mentioned by 7% of the 68 institutions recommending antidepressants for prevention.

Three quarter of hospitals (59/81) recommend psychotherapy as a preventive treatment. On average they suggest it to 62% of their SAD patients. Two fifths recommend continuous psychotherapy, one fifth suggests psychotherapy that lasts up to 30 weeks. Most frequently, behavioural therapy is recommended (see Table 2).

Three quarters (58/81) consider light therapy a viable option for prevention. They recommend it on average to 64% of their SAD patients. One third recommend continuation of light therapy throughout summer to their patients, while nearly half of the departments and hospitals recommend stopping during summer and starting by the end of summer before depressive symptoms occur. Most of the departments and hospitals recommend a light therapy device with 10,000 lux (Table 2).

Nearly half of the participating departments and hospitals (38/81) recommend diet changes. On average they suggest this to 71% of their SAD patients. Most advise a permanent change and advise cutting out caffeine, nicotine, and alcohol consumption, maintaining a balanced diet (e.g. Mediterranean diet), drinking enough fluids, adding vitamin D to their diet and trying to avoid heavy meals in the evening (Table 2).

Half of the hospitals recommend alternative approaches such as yoga, acupuncture, meditation or other relaxation techniques as preventive treatments to half of their SAD patients. Nearly half of the hospitals recommend agomelatine, and only a few hospitals melatonin (9/81), or methylphenidate (4/81, further details Table 2).

We found no differences in recommendations of preventive interventions between countries, regions and type of hospital (Table 3).

**Table 3:** Differences in use of preventive treatment between countries, type of hospital, and region

Subgroups	Use of preventive treatment	Fisher’s *p*-value
Country (*n* = 96)		0.134
Austria (*n* = 18)	94%	
Germany (*n* = 67)	79%	
Switzerland (*n* = 11)	100%	
Type of hospital (*n* = 95)		0.923
University hospital (*n* = 20)	85%	
Teaching hospital (*n* = 22)	82%	
Specialized psychiatric hospital (*n* = 32)	90%	
Psychiatric department in general hospital (*n* = 21)	81%	
Northern vs. southern regions (*n* = 95)		0.261
South (*n* = 47)	89%	
North (*n* = 48)	79%	

n, total number of participating hospitals that answered this question

*SAD* seasonal affective disorder

Comparing different types of preventive treatments between northern and southern regions, showed no statistically significant differences, except for one: 61% of hospitals and departments in northern regions recommend agomelatine, compared to 37% in southern regions (Table 4).

**Table 4:** Differences between type of preventive treatment between northern and southern regions

Type of preventive treatment	North	South	Fisher’s *p*-value
Antidepressants	90% (*n* = 38)	83% (*n* = 40)	0.519
Lifestyle changes	87% (*n* = 37)	88% (*n* = 41)	1.000
Psychotherapy	73% (*n* = 37)	76% (*n* = 41)	0.802
Light Therapy	72% (*n* = 39)	73% (*n* = 40)	1.000
Agomelatine	61% (*n* = 38)	37% (*n* = 41)	0.044^a^
Dietary suggestions	49% (*n* = 37)	50% (*n* = 40)	1.000
Alternative approaches	38% (*n* = 37)	34% (*n* = 41)	0.815
Melatonin	5% (*n* = 37)	17% (*n* = 41)	0.159
Methylphenidate	0% (*n* = 37)	10% (*n* = 41)	0.117

^a^Statistically significant

n, total number of participating hospitals that answered this question

## Discussion

SAD causes a great burden of disease. However, with the exception of our study, no data exists to date in regards to what measures psychiatric hospitals and departments recommend to their patients in order to prevent the onset of new depressive episodes. In the present study, we observed for the first time that most hospitals prefer to suggest preventive measures before the actual onset of SAD, during the months preceding fall/winter. Most hospitals used different types of preventive treatments, indicating that prevention strategies comprise a mix of interventions and not solely one specific treatment option. In addition, a novel observation was made whereby the changes in life style and the use of antidepressants were the most prevalent forms of preventive treatment recommended by hospitals.

The use of preventive treatment is high with 84% of psychiatric hospitals and departments recommending it to patients with a history of SAD, even though clinical practice guidelines do not recommend preventive treatment for SAD [28] and hardly any evidence on efficacy and safety of preventive treatments in SAD patients exist [24–26, 36]. In order to provide psychiatrists with guidance on preventive treatment, controlled studies investigating the efficacy and harm of different preventive interventions and comparing them with each other are needed.

Interventions recommended most frequently for prevention are lifestyle changes, antidepressants, light therapy, and psychotherapy. Respondents reported prescribing a wide variety of antidepressants, although only one (Bupropion XL) is licenced for this indication. One explanation could be that psychiatrists often define it as preventive use to continue acute treatment with antidepressant throughout the summer. In this case they seem to select from all types of antidepressants, probably considering effectiveness and side effects of antidepressants in prior acute treatment periods.

This is in line with another interesting finding, in that the use of acute treatment options for acute SAD is very similar to the use of these interventions as preventive treatment. This could be because psychiatrists and patients are more willing to use an intervention in a symptom-free time as prevention, when this intervention has already proven effective in the acute, depressive phase.

About three quarters used light therapy for acute and preventive treatment of SAD. This agrees with another study that showed that 79% of institutions considered light therapy useful in acute SAD treatment [31].

Comparisons between northern and southern regions revealed no differences in the use of preventive treatments and type of preventive interventions recommended, although higher prevalence of SAD in northern latitude [3–7, 37] would suggest higher demand for preventive treatment. The only difference identified was that agomelatine is prescribed more often in northern regions. This might be because this medication gets reimbursed only in Germany.

There are some limitations to this study: First, not all addressed institutions participated in our survey. The response rate (19%) was moderate. However, response rates around 20% are common in postal and web-based surveys [30]. It is possible that especially institutions that do not treat patients for SAD refrained from participating in our survey, which may have introduced a selection bias. However, we assume that only institutions that treat SAD patients can recommend preventive treatment to them. Therefore, the selection bias likely did not substantially influence our results.

A further shortcoming of this study is that the numbers reported from all respondents are their best estimates and are not based on actual documentation. This trade-off was necessary to make completing the survey feasible within a decent time frame. However, this may have introduced bias, such as heads of departments remembering some types of preventive treatments better than others.

Third, we focused on psychiatric hospitals and departments, not on office-based general practitioners and psychiatrists who might be the first point of contact for many SAD patients. We did not survey general practitioners because studies have shown that SAD is often under- or misdiagnosed in this setting [5]. Goldberg and Bridges [38] assume that this is because it may be difficult for general practitioners to recognize forms of depression that are characterized by atypical symptoms such as weight gain, fatigue and hypersomnia, all of which commonly occur in SAD. However, for future studies it may be interesting to investigate recommendation of preventive strategies by office-based general practitioners and psychiatrists in SAD patients, especially in light of new findings indicating that 60% of depression-related treatment in Germany is provided by general practitioners [39].

## Conclusions

Preventive treatment in patients with a history of SAD is very common in clinical practice in German-speaking countries and a wide variety of treatment options is used. However, the evidence on efficacy and harms of preventive treatment in SAD patients is limited. At the moment, psychiatrists seem to draw conclusions from efficacy of acute treatment of SAD to efficacy of preventive treatment. To assist psychiatrists and patients in decision making and provide guidance, controlled comparative effectiveness studies on preventive treatment are needed. Considering the current lack of evidence, the selection of treatment for preventing winter depression should be strongly based on patient preferences. Therefore studies on patients’ satisfaction and perceived benefit to those interventions are needed.

## Abbreviations

Max: Highest percentage named
Min: Lowest percentage named
n: Total number of hospitals answering this question
SAD: Seasonal affective disorder
XL: Extended release
